# Targeted Nanotechnology in Glioblastoma Multiforme

**DOI:** 10.3389/fphar.2017.00166

**Published:** 2017-03-31

**Authors:** Talita Glaser, Inbo Han, Liquan Wu, Xiang Zeng

**Affiliations:** ^1^Department of Biochemistry, Institute of Chemistry, University of São PauloSão Paulo, Brazil; ^2^Department of Neurosurgery, Spine Center, CHA University, CHA Bundang Medical CenterSeongnam, South Korea; ^3^Department of Neurosurgery, Renmin Hospital of Wuhan UniversityWuhan, China; ^4^Department of Histology and Embryology, Zhongshan School of Medicine, Sun Yat-sen UniversityGuangzhou, China

**Keywords:** cancer stem cell, glioma, nanotechnology, targeted therapy, blood–brain barrier, nanomedicine

## Abstract

Gliomas, and in particular glioblastoma multiforme, are aggressive brain tumors characterized by a poor prognosis and high rates of recurrence. Current treatment strategies are based on open surgery, chemotherapy (temozolomide) and radiotherapy. However, none of these treatments, alone or in combination, are considered effective in managing this devastating disease, resulting in a median survival time of less than 15 months. The efficiency of chemotherapy is mainly compromised by the blood-brain barrier (BBB) that selectively inhibits drugs from infiltrating into the tumor mass. Cancer stem cells (CSCs), with their unique biology and their resistance to both radio- and chemotherapy, compound tumor aggressiveness and increase the chances of treatment failure. Therefore, more effective targeted therapeutic regimens are urgently required. In this article, some well-recognized biological features and biomarkers of this specific subgroup of tumor cells are profiled and new strategies and technologies in nanomedicine that explicitly target CSCs, after circumventing the BBB, are detailed. Major achievements in the development of nanotherapies, such as organic poly(propylene glycol) and poly(ethylene glycol) or inorganic (iron and gold) nanoparticles that can be conjugated to metal ions, liposomes, dendrimers and polymeric micelles, form the main scope of this summary. Moreover, novel biological strategies focused on manipulating gene expression (small interfering RNA and clustered regularly interspaced short palindromic repeats [CRISPR]/CRISPR associated protein 9 [Cas 9] technologies) for cancer therapy are also analyzed. The aim of this review is to analyze the gap between CSC biology and the development of targeted therapies. A better understanding of CSC properties could result in the development of precise nanotherapies to fulfill unmet clinical needs.

## Introduction

Gliomas, demonstrating glial cell characteristics, represent 30% of all brain tumors as described by American Cancer Society (2016). These tumors, especially high-grade gliomas and glioblastoma, grow invasively in the central nervous system and cause discernible neurological symptoms within months with an extremely poor prognosis even after aggressive open surgery combined with adjuvant chemo/radiotherapy. New assumptions incriminate cancer stem cells (CSCs) as a possible cause of tumor treatment resistance. However, the biological nature of these cells is still undetermined (Louis et al., 2007; Westphal and Lamszus, 2011).

The development of new technologies based on nanometer-sized particles (nanotechnology) for cancer treatment has been extensively investigated in the last decade and this approach shows potential for glioma diagnosis and treatment. Unique molecular signatures for each type of tumor have been uncovered recently, because of advances in proteomics and genomics, opening new paths for therapies that specifically target and kill tumor cells (Cruceru et al., 2013).

In this review paper, the challenges in targeting gliomas are highlighted. The concept of CSCs and their biomarkers is introduced initially, and finally, developed nanotechnologies, including some clinical trials, are summarized. Moreover, the application of therapies already used in different fields to glioblastoma multiform (GBM) treatment is proposed, focusing on CSC targeting.

## Clinical Classification And Current Treatment Of Gliomas

Gliomas are brain tumors that resemble normal stromal (glial) cells of the brain, such as astrocytes (astrocytomas), oligodendrocytes (oligodendrogliomas) and ependymal cells (ependymomas). They are a group of oncological diseases for which no cure exists and little progress has been made in order to guarantee a longer life expectancy. Gliomas can diffusely penetrate throughout the brain and are mainly classified according to their morphological resemblance to their respective glial cell types, their cytoarchitecture and their immunohistological marker profile (Louis et al., 2007; Westphal and Lamszus, 2011).

There is also a glioma grading system that distinguishes, astrocytomas, by four World Health Organization (WHO) grades (I, II, III, and IV); and oligodendrogliomas and oligoastrocytomas, by two grades (II and III) (Louis et al., 2007).

The most aggressive and common glioma is glioblastoma (a grade IV astrocytoma). This tumor demonstrates extensive vascular endothelial proliferation, necrosis, high cell density and atypia. It can evolve from a preexisting secondary glioblastoma (low grade astrocytoma), but usually occurs *de novo* (primary glioblastoma) (Westphal and Lamszus, 2011).

Recently, as described in the 2016 WHO report on the central nervous system (CNS), it has been recommended that glioblastomas be divided into IDH-wildtype, IDH-mutant and Nitric oxide synthase (NOS). IDH-wild type (about 90% of cases) is regarded as primary or *de novo* glioblastoma and prevailing in patients over 55 years of age; IDH-mutant (about 10% of cases), corresponds to secondary glioblastoma that preferentially arises in younger patients (Louis et al., 2007); and NOS is reserved for cases in which a full IDH evaluation cannot be performed (Louis et al., 2016).

In the last two decades, glioblastoma treatment using chemotherapy has undergone some changes, such as replacing the use of some alkylating substances like carmustine (BCNU), nimustine (ACNU), and lomustine (CCNU) with temozolomide (TMZ). The alkylating agent groups that have been mostly prescribed in the clinic are: TMZ (8-Carbamoyl-3-methylimidazo (5, 1-d)-1, 2, 3, 5-tetrazin-4(3H)-one) and nitrosoureas (BCNU, ACNU, CCNU – also referred to as CNUs) (Beier et al., 2011).

Temozolomide is rapidly converted into its reactive format, 5-3-(methyl)-1-(triazen-1-yl) imidazole-4-carboxamide, at physiologic pH, causing DNA damage through methylation of the O6-position of guanines, blocking DNA replication and inducing the death of tumor cells (Kaina et al., 1997; Ochs and Kaina, 2000; Roos and Kaina, 2006) or even cell cycle arrest (Hirose et al., 2001).

In contrast, the CNUs alkylate the N3-position of adenine and the N7-position of guanine inducing apoptotic cell death in p53 wildtype cells and necrotic cell death in p53 deficient cells (Fischhaber et al., 1999; Johannessen et al., 2008).

Currently, TMZ, together with radiotherapy and surgical resection, is the most commonly applied glioblastoma treatment. Despite a boost in overall patient survival with TMZ treatment and the low toxicity of TMZ, patient prognosis remains poor. Usually few patients survive longer than 5 years, with a median survival of approximately 14.6 months (Stupp et al., 2005, 2009).

## GBM Stem Cells and Treatment Resistance

The possible cause of GBM chemoresistance is the presence of CSCs. CSCs are tumor cells with stem cell-like properties that reside in GBM and can readily generate both proliferating progenitor-like and differentiated tumor cells amid microenvironment cues (Morokoff et al., 2015). CSCs could be more resistant towards radio- and chemotherapy and survive intensive oncological therapies, leading to tumor recurrence (Modrek et al., 2014). Since GBM is an aggressive tumor, the development of alternative therapies targeting CSCs is urgently needed.

The origin of CSCs can be either mutated embryonic stem cells or downstream progenitors, that may already exist at birth or accumulate over time through mutation (Shipitsin and Polyak, 2008). Recent studies have revealed that the “de-differentiation” of non-CSCs into CSCs can be an alternative mechanism of CSC creation (Safa et al., 2015), suggesting that diverse cell types, from stem cells to their related differentiated progeny, are amenable to oncogenic transformation.

Distinguishing between CSCs and other tumor populations largely lies in the functional multipotency that stem cells demonstrate, i.e., the self-renewal and differentiation to multiple progeny capabilities. Cells that are tumorigenic and can differentiate hierarchically are commonly regarded as CSCs (termed alternatively as glioma stem cells, glioma CSCs, or brain tumor stem cells). Also, CSCs can form sphere-shaped colonies, however, it is not considered as a default feature (Pastrana et al., 2011).

## Biomarkers for Glioma Stem Cells

The CSC hypothesis states that CSCs escape multimodal therapy, causing tumor resistance. Some causes of this resistance could be insufficient drug delivery to CSCs niche or non-specific targeting, since the therapies generally target more differentiated tumor cells. Another premise of this hypothesis is that therapies which efficiently eliminate the CSC fraction of a tumor are able to induce long-term responses and thereby halt tumor progression. The best-described marker for CSCs is CD133, and recently new molecules such as CD15/ stage specific embryonic antigen-1 (SSEA-1) and integrin a6 have been described as novel markers. However, there is not yet a consensus on the optimal markers for CSCs in GBM. CSCs have been isolated from cancer to be analyzed and later used to screen for stem cell-specific biomarkers in tumor cells, particularly surface biomarkers. Cell-surface markers are generally cell membrane-surface antigens to which antitumor drugs can easily bind, consequently increasing the therapeutic efficiency of the drug. Therefore, membrane surface markers are more meaningful than nuclear or cytoplasmic antigens in targeted tumor therapy.

### CD133 and its Limitations

CD133 belongs to the Prominin family, and is also known as Prominin 1, with five transmembrane regions. Singh et al. (Singh et al., 2004) found that 100 CD133 positive cells is enough to induce tumorigenesis in the NOD/SCID mouse brain and whereas 100,000 CD133 negative cells were incapable of tumorigenesis. Subsequently, CD133 has been widely recognized as a biomarker of glioma stem cells.

Although many studies have demonstrated transplanted tumors using CD133+ cells, some researchers have reported on the limitations of CD133 as tumor stem cell marker. CD133+ cells serve as tumor stem cells in many organs, such as brain, lung and colon cancers, but expect for gastric or breast (Su et al., 2015). CD133+ cells only had tumor initiating effects in some glioma cells and were not found in other brain tumors, such as CD15+, CD133- medulloblastomas (Read et al., 2009). Different types of glioblastoma cells derived from different patients can produce CD133+ or CD133- tumor stem cells after serum-free culture *in vitro*, both of whom embrace stem cell features, tumorigenic characteristics and capability of re-generating CD133+ and CD133- cell populations. CD133+ glioma stem cells can differentiate into CD133- tumor cells; CD133- glioma cells injected into nude rats formed tumors containing CD133+ cells (Joo et al., 2008; Wang et al., 2008). Therefore, CD133+ cells are not the only cells with the characteristics of glioma stem cells, and CD133- cells exist as CSCs.

### CD44

Recent studies have demonstrated that some glioma cell subpopulations highly express CD44, a distinctive cell adhesion molecule (Xu et al., 2010). CD44 is a glycoprotein commonly expressed in numerous malignancies (Bradshaw et al., 2016). CD44 knockdown in GBM xenograft models has inhibited tumor cell growth while improving the response to chemotherapy (Y. Xu et al., 2010). CD44 and CD133 are usually co-expressed in GBM spheres (Brown et al., 2015). Collectively, these data suggest that CD44 may be useful as a CSC marker.

### Integrin-α6

Integrin-α6 is a member of the heterodimer integrin family and is a laminin member of the extracellular matrix protein family. Integrin-α6 can be used as a marker of neural stem cells and the expression of integrin-α6 can be used to detect the tumorigenic potential of normal neural stem cells (Corsini and Martin-Villalba, 2010). Integrin-α6 is highly expressed by the glioma stem cell population and can be used to isolate glioma stem cells (Lathia et al., 2010; Velpula et al., 2012). The function of integrin-α6 lies in self-renewal, proliferation, survival and growth of tumor cells *in vitro*, and so it can be used as a regulatory target of tumor growth, while its genetic knockout can reduce tumorigenesis (Lathia et al., 2010).

### CD15

Also known as SSEA-1, it is a carbohydrate antigen on the cell surface. Read et al. (Read et al., 2009) found that tumor cells that are CD15+ and CD133- had the characteristics of tumor stem cells through mouse medulloblastoma experiments. The tumorigenicity of CD15+ cells is 100 times higher than that of CD15- cells in human glioblastoma, where all CD15+ cells were also found to be CD133+, while most CD133+ cells also expressed CD15, suggesting that CD15 is highly likely to be another surface marker of glioblastoma stem cells (Son et al., 2009).

### L1CAM

L1 cell adhesion molecule (L1CAM) belongs to the nerve cell adhesion molecule category and to the type I transmembrane glycoprotein of immunoglobulin super family and is crucial in nervous system development. L1CAM supports the survival and proliferation of CD133+ glioma cells, both *in vitro* and *in vivo*, and can be targeted as CSC-specific marker for precise treatment in malignant gliomas (Bao et al., 2008). L1CAM activates some signaling pathways such as fibroblast growth factor receptor (FGFR) and focal adhesion kinase (FAK) through integrin, increasing the growth and motility of GBM cells in autocrine or/and paracrine manner. These effects can be intervened by using small-molecule inhibitors of FGFR, integrins and FAK (Anderson and Galileo, 2016).

### CD90

Also known as Thy-1, CD90 is a member of the cell adhesion molecule immunoglobulin super family. CD90 has been found on the surfaces of nerve cells, thymocytes, fibroblast subsets, endothelial cells, mesangial cells, and hematopoietic stem cells, suggesting that CD90 is a surface marker in hematopoietic stem cells (Kumar et al., 2016), mesenchymal stem cells (Kimura et al., 2016) and hepatocellular stem cells (Yang et al., 2008). CD90 is overexpressed in GBM and is almost absent in low-grade gliomas or normal brain tissues. All CD133+ glioma cells expressing CD90, and CD90+/CD133+ and CD90+/CD133- cells have the same self-renewal ability, indicating that CD133+ glioma stem cells may be a subtype of CD90+ glioma cells (He et al., 2012). In addition, CD90+ cells were also found in glioma peritumoral vessels (Inoue et al., 2016). Therefore, CD90 can be used as a prognostic index of glioma, a marker of glioma stem cells and an indicator of glioma angiogenesis as well.

### A2B5

A2B5 is a ganglioside on the surface of the glial precursor cell membrane. Ogden et al. (Ogden et al., 2008) detected more A2B5+ cells than CD133+ cells in glioblastoma samples, and CD133+ cells were rarely detected. The cells were screened and sorted using flow cytometry, and sequential culture of A2B5+/CD133- and A2B5+/CD133+ cells showed stem cell proliferative activity while that of A2B5-/CD133- cells did not. Tchoghandjian et al. (Tchoghandjian et al., 2010) confirmed that A2B5+/CD133- and A2B5+/CD133+ cells could form tumor stem cell spheres, while A2B5-/CD133- cells could not. These studies also show that CD133- cell populations still contain cells with stem cell activity, and A2B5+ cells may be one type of such stem cells. CD133-/A2B5+ glioma-initiating cells possess a strong migratory and invasive capacity; these cells may be an important subpopulation with high invasive potential in GBM (Sun et al., 2015).

Recently, some typically expressed embryonic stem cells markers have been considered as the markers for tumor-initiating cells, such as c-Myc, SOX2, and OCT-4. These markers could be useful as a tool to identify and isolate CSCs (Ignatova et al., 2002). Moreover, Nestin, OCT-4, NANOG, SOX2, c-Myc, and KLF4 have been described as key players in the transcriptional regulation of glioblastoma CSCs (Ignatova et al., 2002; Yang et al., 2008; Guo et al., 2011; Zhu et al., 2014).

## Application of Nanotherapies in GBM

Besides drug discovery, the delivery of drugs to the brain is a major challenge in treating CNS diseases. Invasive procedures like tumor resection are not always effective for cancer treatment and are extremely complicated and delicate. A possible alternative to overcome this issue is to use systemic delivery; however, the blood–brain barrier (BBB) is an obstacle because of its low permeability, requiring higher doses of drugs, which causes increased side effects. The BBB inhibits the delivery of therapeutic agents to the CNS and prevents a large number of drugs, including antibiotics, antineoplastic agents, and neuropeptides, in passing through the endothelial capillaries to the brain (Fiandaca et al., 2011; Aryal et al., 2014; Pardridge, 2014). Safe disruption or loosening of the BBB is highly important to deliver drugs into brain niches. Successful delivery of drugs can be achieved through BBB disruption using ultrasound in intra-arterial infusion therapy. This allows both chemotherapeutic agents and antibodies to bypass the BBB (Kuittinen et al., 2013). In addition, K^+^ (Ca) channels have been identified as potential targets for modulation of BBB permeability in brain tumors by assisting the formation of pinocytic vesicles of drugs (Ningaraj et al., 2003). Moreover, tumor drug delivery can be enhanced if they are injected into the brain along with a vasodilator, such as bradykinin, nitric oxide donors or agonists of soluble guanylate cyclase, and calcium dependent potassium K^+^ (Ca) channels. Furthermore, cerebral blood flow could be modulated and the therapeutic efficacy was augmented after applying a nitric oxide donor which selectively open the blood tumor barrier in rats with intracerebral C6 gliomas (Fross et al., 1991; Weyerbrock et al., 2003, 2011; Black and Ningaraj, 2004).

Aiming to enhance transport through or bypass the BBB, many research groups have been developing new nanotechnologies to overcome these obstacles. Many biochemical modifications of drugs and drug nanocarriers have been developed, enabling local delivery of high doses while avoiding systemic exposure. In this review section, BBB properties and recently discovered nanotechnologies that allow systemic drug delivery for CNS cancer therapy are discussed.

## The Blood–Brain Barrier

**FIGURE 1:**
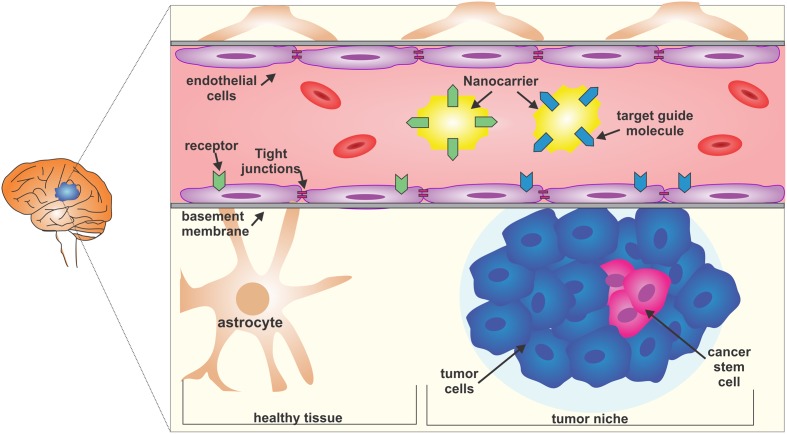
**The blood-brain barrier (BBB) and the glioblastoma multiform (GBM) niche.** The BBB is selective and restrictive to a variety of molecules. Endothelial cells and the basement membrane, together with strong lateral tight junctions, maintain the selective permeability. A possible strategy to reach the glioma core is to use nanocarriers coupled with target guiding molecules that, for example, bind to the membrane receptors of both tumor niche infiltrated BBB or healthy BBB, and which carry nanomedicines. Glioblastoma is composed of heterogeneous cell populations and the cancer stem cells are responsible for treatment resistance.

The BBB is a barrier that presents selective permeability carried out by endothelial cells lining the lumen of brain capillaries, which lack pinocytosis and fenestrations because of the presence of tight junction complexes (Eichler et al., 2011; Chacko et al., 2013; Papademetriou and Porter, 2015). In addition to tight junction complexes, the BBB degrade drugs preventing them to reach the target location due to drug metabolizing enzymes presence, besides the existence of active efflux transporters (AETs) that cargo drugs back to the blood and enzymes that metabolize the drugs before their releasing to the destination. (Regina et al., 2001; Ohtsuki and Terasaki, 2007; Papademetriou and Porter, 2015) (**Figure 1**). The tight junctions in the BBB are mainly composed of claudins and occludins (Nitta et al., 2003; Abbott et al., 2010; Haseloff et al., 2014). Claudin 5 is critical for the restriction of small molecules (<800 daltons) and the loss of some claudins, like claudin 3, is related to the increased BBB permeability in tumor vasculature and autoimmune encephalomyelitis (Wolburg et al., 2003). In contrast, the BBB remains intact in infiltrating gliomas or micrometastatic tumors, indicating that it is crucial to modulate the BBB permeability in these regions.

Transport across the BBB is selective for molecules smaller than 12 nm and is finely regulated; there are mainly two types of transport, carrier-mediated transport (CMT) and receptor-mediated transport (RMT) (Papademetriou and Porter, 2015) (**Figure 1**).

### Carrier-Mediated Transport

The transport of energy production molecules like glucose and lactate, nucleosides, and ions through the cell membrane by facilitated or active transport can be mediated by CMT. In addition, CMT aids the clearance of neurotoxic substances, metabolites of brain function, and neurotransmitters, through AETs (Ohtsuki and Terasaki, 2007; Sanchez-Covarrubias et al., 2014; Papademetriou and Porter, 2015).

The biochemical modification of small molecules enables changes in some parameters like solubility, stability, lipophilicity, and recognition by AETs. Redesign of drug aiming to improve the recognition by CMT and transportation through BBB can be achieved by coupling the drug to a regular CMT substrate. The molecular structure of the drug should mimic that of the endogenous CMT substrate (e.g., sugars, amino acids, nucleosides) with pharmacologic activity preserved, but preferably not affect CMT function to avoid possible side effects (Misra et al., 2003; Papademetriou and Porter, 2015).

### Receptor-Mediated Transport

In contrast to CMT, RMT promotes the permeability of some macromolecules into the brain, such as lipoproteins, hormones, nutrients and growth factors (Papademetriou and Porter, 2015). The RMT process is mediated by the binding of the molecule to a cell-surface receptor that presents in endothelial cells on the luminal surface, following endocytosis and transportation of vesicles to the destination, and sequential exocytosis of the vesicle to the extravascular space (Abbott et al., 2010; Georgieva et al., 2014; Papademetriou and Porter, 2015).

The approach targeting RMT requires the involvement of a specific ligand (e.g., an antibody or antibody fragment, synthetic peptide, natural ligand), which has affinity for an endocytic receptor expressed on the endothelial cell surface, to the chemotherapeutic drug or to a drug-loaded nanocarrier. Binding to the targeted receptor induces intracellular signaling cascades mediating invagination and formation of membrane-bound vesicles in the cell interior, and then intracellular vesicular trafficking transport to the abluminal endothelial plasma membrane (Abbott et al., 2010; Georgieva et al., 2014; Papademetriou and Porter, 2015).

## Nanocarriers

**FIGURE 2:**
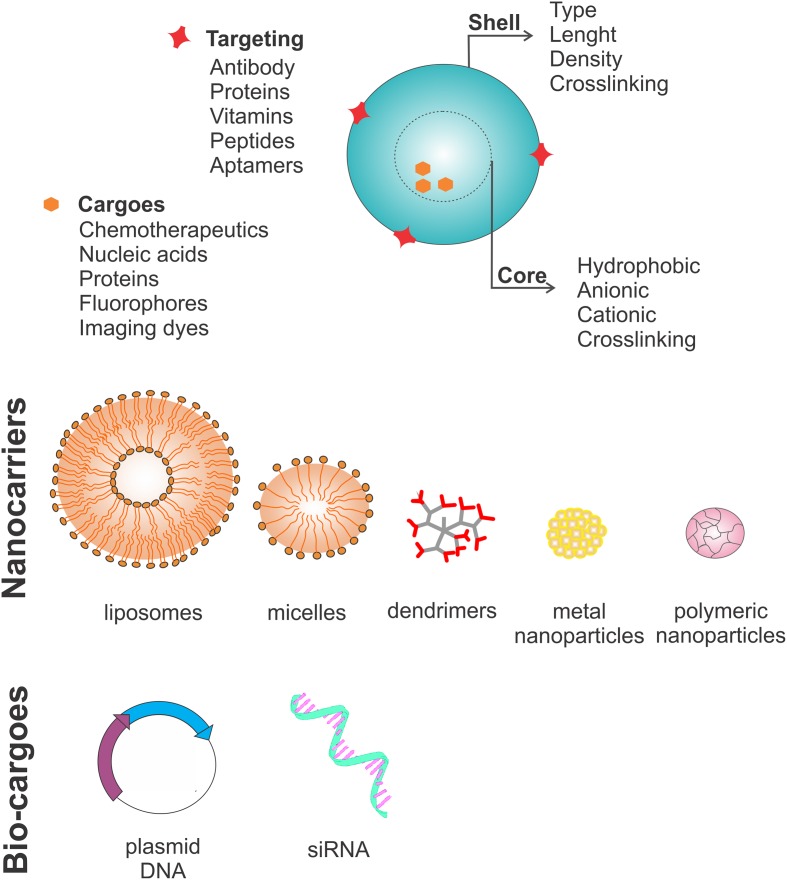
**Nanocarrier characteristics.** Nanocarriers have four main features: a shell that can vary in type, length, density and crosslinking molecules; a core, which can be hydrophobic, anionic or cationic depending on which crosslinked molecule needs to be carried; surface targeting molecules which can be antibodies, proteins, vitamins, peptides and aptamers; and lastly the cargo, which can be chemotherapeutics, nucleic acids, proteins, fluorophores or other imaging dyes. Usually nanocarriers are divided into five subtypes: liposomes (lipid bilayer structures), polymeric micelles (lipid monolayers), dendrimers (highly branched structures), and nanoparticles (organic or inorganic). Recently, new strategies have focused on carrying bio-cargoes, such as plasmids coding for proteins involved in programmed cell death or agents to silence the genes important for the cancer stem cell survival through genetic knockout using the CRISPR/CAS9 system or genetic knockdown using siRNAs.

The discussion of nanosystems in this review mainly focuses on liposomes, polymeric nanoparticles, solid lipid nanoparticles, polymeric micelles and dendrimers as carriers (**Figure 2**).

### Liposomes

Lipid-bilayer vesicles, namely liposomes, are popular drug systems for delivery due to their easy preparation, their encapsulation capability of a wide array of drugs, their biocompatibility, efficiency, non-immunogenicity, enhanced solubility of chemotherapeutic agents, and commercial availability. The clearance of liposomes by macrophages is relatively fast, so modifications of the liposome surface or size can extend their circulation time. Specificity for the nervous system is possible by coupling liposomes to aptamers or monoclonal antibodies against transferrin receptors (OX-26), glial fibrillary acidic proteins or the insulin receptor (Kanai et al., 2014). The use of liposomes for gene delivery has been demonstrated by injecting liposomes carrying a plasmid coding for the green fluorescence protein in rats. Also, in tumor therapy, liposomes carrying small interfering RNA (siRNA) have been deployed, while the diesteryl phosphoethanolamine poly carboxybetaine lipid which promotes endosomal/lysosomal escape was developed for systemic delivery of siRNA (Dai et al., 2014; Ozpolat et al., 2014).

Furthermore, trafficking cargo across the BBB is improved when using nanocarriers that target CMT. For example, liposomes targeting glucose transporter 1 (GLUT1) enhanced transport of daunorubicin (Ying et al., 2010), while doxorubicin delivery to the brain was 4.8-fold enhanced after equipped with liposomes that targeted glutathione transporters (2B3–101). This approach is particular suitable for small molecules delivery rather than that of large ones. In another study, 2B3–101 was reported as reaching clinical trials and this will be further detailed along this review (Birngruber et al., 2014; Papademetriou and Porter, 2015).

### Nanoparticles (NPs)

Nanoparticles (NPs) have also been widely studied, because of their high drug-loading capacity and protection against chemical and enzymatic degradation. NPs have enormous medical potential and have emerged as a major tool in nanomedicine, compared with conventional drug delivery methods. NPs are solid colloidal particles made of polymers ranging from 1 to 1000 nm, and are divided in two types, nanospheres and nanocapsules (Couvreur et al., 2002). An interesting application of NPs is the magnetic format of NPs that are made of a magnetic core of iron oxide or magnetite and a biocompatible covering shell of dextran or starch, to be distributed through an organism that is exposed to a localized magnetic field. *In vivo* GBM models have shown that magnetic NPs are promising. Detailed reviews concerning NP applications have already been published (Laquintana et al., 2009; Upadhyay, 2014).

### Polymeric Micelles

Polymeric micelles range from 10 to 100 nm and have a core-shell architecture like NPs. They spontaneously self-assemble in aqueous solutions at concentrations higher than a threshold concentration termed the critical micelle concentration. The core is constructed mainly by hydrophobic polymer parts such as poly(caprolactone), poly (propylene glycol) (PPG), or poly(D,L-lactide), together with a hydrophilic shell made of poly(ethylene glycol) (PEG). Pluronic micelles (PEG-PPG-PEG) have emerged as good candidates for brain therapy, since they can easily cross the BBB and inhibit drug efflux. Micelles carrying paclitaxel were able to increase the toxicity of the chemotherapeutic drug in a LN18 human glioblastoma cell line (Liu et al., 2008; Laquintana et al., 2009).

### Dendrimers

Dendrimers are highly branched polymer molecules smaller than 12 nm. Conjugation to dendrimers confers enhanced delivery across the BBB, which in polyether-copolyester dendrimers loaded with methotrexate and D-glucosamine and tested against avascular glioma spheroids resulted in increased methotrexate potency. After a week, dendrimers do not affect the viability of neural cells nor induce local microglia activation even at submicromolar range of concentration. A better understanding of dendrimer distribution patterns may facilitate the design of nanomaterials for future clinical applications (Laquintana et al., 2009).

### Metal Particles

Metal particles have been studied extensively because it has been demonstrated that they enhance the susceptibility of tumor tissues to injury induced by radiation exposure, and are therefore a promising candidate for nanomedicine. Application of gold NPs prior to radiation produced distinctive DNA damage in tumors and improved the survival of tumor bearing animals (Joo et al., 2008; Bobyk et al., 2013). Previous research has suggested that the enhanced radiosensitization effects were led by low-energy electrons emission from gold particles and in a dose-dependent maner (Zheng et al., 2008). Similarly, the radiosensitization effect of silver NPs is also attributed to their interactions with the DNA repair system, which eventually leads to the arrest of DNA duplication and cell apoptosis (Xu et al., 2009). After irradiation, titanium dioxide (TiO2) induces tumor cell death by increasing the production of free radicals. The amount of reactive oxygen species generated is dose-dependent on the amount of TiO2 applied as a radiosensitizer when the cells are exposed in X-rays (Park et al., 2008).

## Emerging Strategies Using Nanocarriers

### Hyperthermia

Hyperthermic treatment strategies use a magnetic medium such as thermoseeds and magnetic NPs to apply moderate heating in a specific area of the organ where the tumor is located. The combination of carbon nanotubes (CNTs) with near-infrared radiation (NIR) was effective in debulking a tumor in rats, leading to tumor shrinkage without recurrence. Furthermore, this protocol could eliminate glioma CSCs, both drug-sensitive and drug-resistant glioma cells due to the broad-spectrum absorption of CNTs by gliomas. In contrast, normal cells were merely affected, demonstrating the lower uptake of CNTs (Santos et al., 2014). Hyperthermia in glioma treatment remains controversial because it is technically difficult to impose a lethal dose of heat to all cell populations within the glioma mass. The heterogeneous response to different grades of hyperthermia may change the biological nature of the surviving tumor cells. For example, following moderate thermal preconditioning human glioma cell lines demonstrate increased proliferation *in vitro* and aberrant aggressiveness in a xenograft model. The transient increase in growth of the CD133 subtype of gliomas after thermal preconditioning indicates that there might be a compensation for the loss of the thermal sensitive sub-population (Zeng et al., 2016).

To further increase selectivity for CSCs, antibodies against the CD133 surface marker can be employed as a targeting moiety. Photothermal therapy using single-walled carbon nanotubes (SWNTs) conjugated with anti-CD133 antibodies (CDSWNTs) produced a targeted lysis of CD133+ GBM CSCs, while CD133- GBM cells remained intact *in vitro*. A discernible shrinkage of tumor after subcutaneous NIR laser irradiation following CDSWNT administration in this particular ectopic GBM tumor model (Wang et al., 2011). NIR photoimmunotherapy, employing a monoclonal CD133 antibody (mAb) conjugated to an IR700 phototoxic phthalocyanine dye, permitted a spatiotemporally controlled elimination of tumor cells through specific image guidance. Rapid cell death was observed after CD133 mAb intravenous administration followed by harmless NIR light applied through the intact skull. This proof of principle study offers a promising theranostic agent that can be applied in intraoperative imaging or histopathological evaluation to define the tumor borders, as well as eradication of CSCs specifically and efficiently (Jing et al., 2016).

### Antitumor Antibiotics

Antitumor antibiotics are a form of chemotherapeutic that interferes with DNA and slows or stops cancer cells from multiplying. Antitumor antibiotics demonstrate promise in treating gliomas. For example, doxorubicin (trade name: Adriamycin^®^), daunorubicin (trade name: Cerubidine^®^), and bleomycin (trade name: Blenoxane^®^), show powerful anticancer activity against gliomas cells *in vitro*. Their efficacy *in vivo* was reported to be poor, which was largely attributed to their inability to penetrate the BBB (von Holst et al., 1990). However, once these antibiotics are encapsulated in PEGylated liposomes (for example, Doxil^®^ is a PEGylated form of liposomal doxorubicin), the prolonged survival of treated animals is observed following an enhanced local antitumor effect (Sharma et al., 1997). Overall, the antitumor effects of liposomal doxorubicin, daunorubicin, or bleomycin have been unsatisfactory against glioma in patients (Fabel et al., 2001; Fiorillo et al., 2004). Further, to promote the efficacy of liposomal formulations against brain tumors, more effective drug-delivery strategies are clearly in need. For example, the combination of ultrasound-induced microbubbles, which create transient local BBB permeability, with liposomal doxorubicin has been reported to have a significant antitumor effect (Aryal et al., 2013, 2015).

### Engineering of Cell Genome

Recently, the advance of new technologies that facilitate the engineering of the cell genome, like clustered regularly interspaced short palindromic repeats (CRISPR)/Cas 9 and silencing RNA, has provided new methods to deliver nucleic acids to the brain, and in particular for glioma treatment. For this purpose, positively charged and degradable polymers, including chitosan, poly(beta-amino esters), poly(amidoamines), and many other cationic polymers have been used, because of their cationic nature, which allows complexation with negatively charged molecules like DNA or RNA. Inorganic NPs are better applied for imaging and drug delivery purposes, because their synthesis is easily tunable and reproducible (Cardoso et al., 2007; Tzeng and Green, 2013). Some examples are injectable superparamagnetic iron oxide NPs, which are used as contrast agents for magnetic resonance imaging, and gold NPs that are used to carry a conjugated drug. Coated spherical gold NPs carrying a highly oriented layer of siRNA are well protected from nuclease degradation and provide highly efficient knockdown.

Liposomes have also been used to deliver the *IFN-*β gene in mouse models of glioma, resulting in immune response induction and reduced tumor growth. Five malignant glioma patients were treated using liposomes carrying the *IFN-*β gene in a pilot clinical trial and four patients showed > 50% tumor reduction or stable disease (Yoshida et al., 2004). Moreover, since Apo2L/tumor necrosis factor-related apoptosis inducing ligand (TRAIL) is fairly specific for cancer cells, a TRAIL plasmid encapsulated in PEG-conjugated PLA NPs (<120 nm) was injected intravenously and caused an increased median survival time (Hawkins, 2004; Lu et al., 2006).

To avoid GBM recurrence, the protein product of the delivered gene should be designed to be active in CSCs. In addition, the construct can be under the control of a cancer-specific promoter, such as survivin or PEG3, to ensure that healthy cells are not transfected or transduced (Su et al., 2005; Van Houdt et al., 2006). The delivery of miRNAs, such as miR-124 and miR-137, can induce terminal differentiation and cell death in murine CSCs *in vitro* (Silber et al., 2008). Moreover, Gangemi et al. used an shRNA-expressing plasmid in a retroviral vector for *in vitro* knockdown of SOX2, leading to inhibited CSC proliferation, self-renewal, and tumor-initiating capacity (Gangemi et al., 2009).

Recently, modified siRNAs have been developed that are protected from nuclease degradation and can be readily taken up into cells. This type of modification allows researchers to focus on developing engineered NPs with a prolonged circulation time and site-specific delivery, instead of siRNA protection, thus accelerating clinical translation.

## Clinical Trials

Few clinical trials using nanotherapies to target GBM have been conducted; this review focuses on glioma treatment, and the information about these clinical trials is summarized in **Table 1**.

**Table 1:** Clinical studies for nanomedicine-based glioma therapy.

Agent/Trade name	Formulation/Composition	Indication/Consequence
NANOTHERM	Iron oxide nanoparticles	Magnetic hyperthermia plus radiotherapy with Nanotherm for the treatment of glioblastoma in 14 patients (Maier-Hauff et al., 2007).
		Hyperthermia plus radiotherapy with Nanotherm for the treatment of glioblastoma in 60 patients (Maier-Hauff et al., 2011). Average survival following first recurrence: 13.2 months compared with 6 months with conventional treatments.
IFN-β	*IFNB* gene therapy via cationic liposomes	A pilot clinical trial of *IFNB* gene therapy to demonstrate its feasibility and safety in glioma treatment (Yoshida et al., 2004).
		Phase I clinical trial of *IFNB* gene therapy for glioma. In histological examinations of autopsy samples many tumor cells showed necrotic changes, and immunohistochemistry identified numerous CD8+ lymphocytes and macrophages infiltrating the tumor and surrounding tissues, while CD34-immunoreactive vessels were notably decreased in the vector-injected brain (Wakabayashi et al., 2008).
INTERLEUKIN 12	Replication-disabled Semliki Forest viral vector carrying the human interleukin 12 (*IL-12*) gene and encapsulated in cationic liposomes (LSFV-IL12)	This was a phase I/II clinical study in adult patients with recurrent GBM which was aimed at evaluating the biological safety, maximum tolerated dose, and antitumor efficacy of LSFV-IL12 (Ren et al., 2003).
DAUNORUBICIN	Liposome	DaunoXome, a liposome formulation of daunorubicin, achieved and maintained potentially cytotoxic levels in glioblastoma for a long time in association with low-level systemic exposure (Zucchetti et al., 1999).
		High concentrations of daunorubicin and daunorubicinol were found in malignant gliomas after systemic administration of liposomal daunorubicin (Albrecht et al., 2001). A combination of liposomal daunorubicin and carboplatin plus etoposide produced a major response and the 29 month progression-free survival was 38% with little and transient hematological toxicity (Fiorillo et al., 2004).
DOXORUBICIN	Liposome	Stabilization of the disease was observed in 54% (7/13) of patients. Partial response and complete response were not observed. Median time-to-progression was 11 weeks. Progression free survival at 12 months was 15%. Median overall survival (OS) after liposomal doxorubicin therapy was 40.0 weeks, whereas the median OS after diagnosis reached 20.0 months (87.0 weeks). Liposomal doxorubicin was well tolerated, with the main side effects being palmoplantar erythrodysesthesia occurring in 38% of patients and myelotoxicity (World Health Organization grade 3–4) in 31% of patients (Fabel et al., 2001). The investigated combination was tolerable and feasible, but neither the addition of PEG-doxorubicin nor the prolonged administration of temozolomide resulted in a meaningful improvement of the patient outcomes (Beier et al., 2009).
		A phase II trial with 40 patients using a combination of temozolomide and pegylated liposomal doxorubicin. Treatment was well tolerated but did not add significant clinical benefit regarding 6-month progression free survival and overall survival (Ananda et al., 2011).
P53	Liposomes encapsulating a normal human wild-type p53 DNA sequence in a plasmid backbone	Phase II Study of Combined Temozolomide and Targeted P53 Gene Therapy (SGT-53) for the treatment of patients with recurrent glioblastoma. This study is currently recruiting participants.
5-FLUOROURACIL	Injectable 5-fluorouracil -releasing microspheres	Phase II study with a total of 95 patients. Safety was acceptable but overall survival was not significantly improved (Menei et al., 2005).


Ang-1005 (also named GRN-1005) was designed to circumvent BBB under several clinical trials. Ang-1005 is conjugated to paclitaxel and to the RMT ligand angiopep-2 that targets LRP1. In a phase I trial, the drug tolerance of maximum dose of 650 mg/m^2^ was shown. A pharmacokinetics and tumor resections analysis proved that Ang-1005 kept intact in blood plasma so as to remain sufficient concentrations for cytotoxicity when approaching tumor samples (Thomas et al., 2009; Papademetriou and Porter, 2015; Regina et al., 2015).

To the best of our knowledge, nanocarrier-based RMT-targeting strategies in GBM treatment have very limit clinical trial outcomes. It has been described that PEGylated liposomal doxorubicin without RMT-targeting was evaluated in phase I studies in GBM patients, showing no improvements in progression nor survival (Papademetriou and Porter, 2015). In Phase I/II clinical trials, solid tumors and metastatic brain cancer or malignant recurrent glioma patients were treated with 2B3–101 encapsulated by a PEGylated liposomal doxorubicin nanocarrier employing glutathione to target glutathione transporters (CMT-based targeting) (Birngruber et al., 2014).

SGT-53 is a nanocarrier composed of cationic liposomes that encapsulate a plasmid for the *p53* tumor suppressor, and which displays scFv-targeting TfR. One phase II clinical trial of SGT-53 is to combine it with TMZ for patients with recurrent malignant gliomas, aiming to evaluate tumor cells death after accumulation of the drugs, anti-tumor efficacy, safety and overall survival (Yu et al., 2004; Senzer et al., 2013).

Some trials are now using gene-silencing therapies, including siRNA coupled to D3 and D5 polylysine dendrimers and melittin-grafted HPMA oligolysine-based copolymers, for intravenous, intracerebroventricular, or intranasal administration to the CNS. A nanoliposomal formulation of irinotecan (CPT-11) is also in phase I trials for glioma (Krauze et al., 2007).

Moreover, magnetically induced hyperthermia, which uses a magnetic medium such as thermoseeds and magnetic NPs to produce moderate heating in a specific area of the organ where the tumor is located, is under investigation for malignant glioma, prostatic cancer, metastatic bone tumors and some other malignant tumors. Thermoseed magnetic induction of hyperthermia for the treatment of brain tumors was first reported by Kida et al. in 1990. A Fe-Pt alloy thermoseed with a length of 15–20 mm, a diameter of 1.8 mm and a Curie point of 68–69°C was used for seven cases of metastatic brain tumor two to three times a week, with the tumor tissues reaching 44–46°C during the treatment. This resulted in two cases of complete response and one case of partial response. Kobayashi et al. (1991) used a thermoseed with a Curie point of 68°C for the treatment of 23 patients with brain tumors, and reported an overall response rate of 34.8% (O’Reilly and Hynynen, 2012; Luo et al., 2014).

PLA is a biodegradable and hydrophobic polymer that can be used as a carrier for hydrophobic chemical drugs for anti-tumor research. Monomethoxy poly(ethylene glycol)-block-poly(D, L-lactide) loaded with paclitaxel to form Genexol^®^-PM has been trialed clinically and is now commercially available for the treatment of breast cancer, ovarian cancer, and non-small cell lung cancer (Kim et al., 2007; Lee et al., 2008). Jun Chen et al. used PEG-PLA as a paclitaxel delivery carrier. The NPs were coupled with the tLyp-1 peptide, which has a high affinity for neuropilin to target both glioma cells and endothelial cells. The tLyp-1-conjugated NPs showed greater penetration in C6 glioma spheroids and enhanced drug access into solid tumors and prolonged survival time to 37 days in intracranial C6 glioma mice, compared with approximately 20 days in controls. However, smart structural design and modification are required for the proper degradation rate of these bioactive materials (Hu et al., 2013).

## Conclusion

In summary, elucidating the biological nature of CSCs offers a new strategy for targeted cancer therapy. Interdisciplinary efforts to develop new nanocarriers that can bypass the BBB, protect the drug from being degraded, and that are specific for tumor cells or CSCs are ongoing. Some groups prefer to focus on developing new drugs that can efficiently kill CSCs, which are responsible for treatment resistance and a poor prognosis in glioblastoma, while some research groups are using modern and pioneering molecular biology tools, such as CRISPR/Cas 9 and siRNA.

**FIGURE 3:**
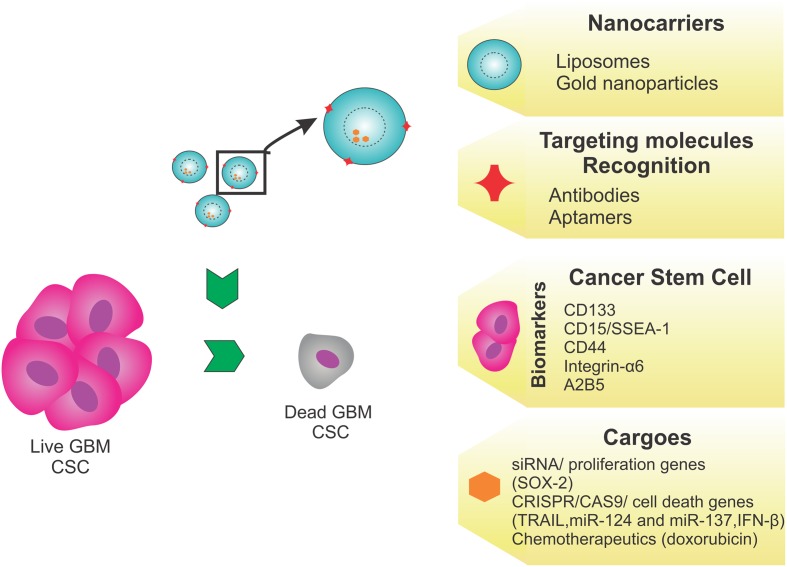
**Proposed strategy.** This proposed therapeutic strategy targeting the GBM cancer stem cells (CSCs) as a novel treatment, would use liposomes as nanocarriers, because they can shield and carry molecules of different sizes and charges. Liposomes, with a shell coated using aptamers or antibodies specific to CSC markers, such as CD133, CD15, CD44, integrin-α6, or A2B5, would carry antitumor antibiotics (doxorubicin) or genome editing tools such as SOX2, TRAIL, miR-124, miR-137, and IFN-β, to modulate tumor survival/death gene expression. Alternatively, the use of gold nanoparticles targeting brain markers, like glial fibrillary acidic protein, is recommended to bypass the BBB and deliver genome editing tools.

To develop a novel treatment based on targeting CSCs, an effective strategy should use liposomes as nanocarriers, because of their ability to shield and carry molecules of different sizes and charges. These liposomes should have a shell coated with aptamers or antibodies specific for CSC markers such as CD133, and would carry antitumor antibiotics (doxorubicin) or genome editing tools that would modulate the expression of genes important for tumor survival, such as *SOX-2*. Another possibility is the use of gold NPs targeting brain markers, such as glial fibrillary acidic protein, to facilitate brain penetration, and deliver siRNA to knockdown tumor survival and proliferation genes (**Figure 3**).

Finally, some clinical trials have succeeded in testing new nanotechnologies that may become available to patients in the near future.

## Author Contributions

TG, IH, LW, and XZ summarized the literature and drafted the manuscript. TG, XZ, and LW revised and edited the manuscript. XZ and LW supervised the work. TG and XZ initiated, finalized, and submitted the manuscript.

## Conflict of Interest Statement

The authors declare that the research was conducted in the absence of any commercial or financial relationships that could be construed as a potential conflict of interest.
